# Integrating distribution kinetics and toxicodynamics to assess repeat dose neurotoxicity *in vitro* using human BrainSpheres: a case study on amiodarone

**DOI:** 10.3389/fphar.2023.1248882

**Published:** 2023-09-06

**Authors:** Carolina Nunes, Susana Proença, Giovanna Ambrosini, David Pamies, Aurélien Thomas, Nynke I. Kramer, Marie-Gabrielle Zurich

**Affiliations:** ^1^ Department of Biomedical Sciences, University of Lausanne, Lausanne, Switzerland; ^2^ Swiss Centre for Applied Human Toxicology (SCAHT), Basel, Switzerland; ^3^ Institute for Risk Assessment Sciences, Utrecht University, Utrecht, Netherlands; ^4^ Toxicology Division, Wageningen University, Wageningen, Netherlands; ^5^ Bioinformatics Competence Center, Ecole Polytechnique Fédérale de Lausanne, Lausanne, Switzerland; ^6^ Bioinformatics Competence Center, University of Lausanne, Lausanne, Switzerland; ^7^ Unit of Forensic Toxicology and Chemistry, CURML, Lausanne and Geneva University Hospitals, Geneva, Switzerland; ^8^ Faculty Unit of Toxicology, CURML, Faculty of Biology and Medicine, University of Lausanne, Lausanne, Switzerland

**Keywords:** neurotoxicity, *in vitro* distribution kinetics, hiPSC, lipid metabolism, spheroid, *in silico* modeling, microphysiological system

## Abstract

For ethical, economical, and scientific reasons, animal experimentation, used to evaluate the potential neurotoxicity of chemicals before their release in the market, needs to be replaced by new approach methodologies. To illustrate the use of new approach methodologies, the human induced pluripotent stem cell-derived 3D model BrainSpheres was acutely (48 h) or repeatedly (7 days) exposed to amiodarone (0.625–15 µM), a lipophilic antiarrhythmic drug reported to have deleterious effects on the nervous system. Neurotoxicity was assessed using transcriptomics, the immunohistochemistry of cell type-specific markers, and real-time reverse transcription–polymerase chain reaction for various genes involved in the lipid metabolism. By integrating distribution kinetics modeling with neurotoxicity readouts, we show that the observed time- and concentration-dependent increase in the neurotoxic effects of amiodarone is driven by the cellular accumulation of amiodarone after repeated dosing. The development of a compartmental *in vitro* distribution kinetics model allowed us to predict the change in cell-associated concentrations in BrainSpheres with time and for different exposure scenarios. The results suggest that human cells are intrinsically more sensitive to amiodarone than rodent cells. Amiodarone-induced regulation of lipid metabolism genes was observed in brain cells for the first time. Astrocytes appeared to be the most sensitive human brain cell type *in vitro*. In conclusion, assessing readouts at different molecular levels after the repeat dosing of human induced pluripotent stem cell-derived BrainSpheres in combination with the compartmental modeling of *in vitro* kinetics provides a mechanistic means to assess neurotoxicity pathways and refine chemical safety assessment for humans.

## 1 Introduction

The brain is structurally and functionally very complex. It is particularly susceptible to toxic insults due to its very low regeneration abilities after damage. In spite of this vulnerability, specific neurotoxicity studies are not systematically conducted in the safety evaluation of chemicals, potentially leading to the exposure of the public to chemicals hazardous to the brain. It has to be noted, however, that a preliminary neurotoxicity evaluation is performed by the histopathological examination of the representative regions of the brain, like the cerebrum, cerebellum, and medulla/pons, for chemicals produced in quantities above 10 tons per year. If neurotoxicity is specifically evaluated, it is generally carried out on rodents. However, the structure and function of a rodent brain is so notably different from a human’s that the relevance of these tests to assess chemical safety in humans is questionable (Bal-Price et al., 2010). In addition, the increasing onset of neuronal disorders and neurodegenerative diseases, linked to the aging of the population, points to a clear demand for new drugs that are active on the nervous system. Although drugs are not under the same regulation as chemicals, their safety has to be assessed in the early phases of drug development (Zheng and Chen, 2022), extending the list of compounds that will require a neurotoxicity evaluation. To overcome the difficulty of testing such a large number of chemicals and drugs, there is a general consensus that animal testing needs to be replaced by a combination of *in vitro* and *in silico* approaches (Bal-Price et al., 2010; Anadón et al., 2014; Fischer et al., 2020; Caloni et al., 2022). Recent advances in human test models, including 3D models, on-a-chip technology and analytical techniques (Pamies et al., 2017; Harrill et al., 2021; Olesti et al., 2021; Castiglione et al., 2022; Parmentier et al., 2023), in combination with adverse outcome pathways (AOPs) (Tollefsen et al., 2014; Villeneuve et al., 2014), *in vitro* distribution kinetics modeling (Pomponio et al., 2015a), and physiologically based kinetic (PBK) modeling for quantitative *in vitro* to *in vivo* extrapolation (QIVIVE) (Kasteel et al., 2021; Noorlander et al., 2022), provide us with a promising new toolbox of new approach methodologies (NAMs) for the 3Rs-based neurotoxicity testing approach or reducing, refining, and replacing (3Rs) animal-based toxicity tests (Anson et al., 2011; Steimberg et al., 2020).

Human induced pluripotent stem cells (hiPSCs) can be differentiated into almost all cell types and can be obtained from specific patient populations to assess interindividual sensitivities to toxic insults. The neurotoxic potencies of chemicals have already been assessed in hiPSC-differentiated neuroprogenitor cells (NPCs), neurons, and glial cells (Druwe et al., 2015; Pei et al., 2016; Ryan et al., 2016; Pistollato et al., 2017). However, not surprisingly, it has been shown that a given brain cell type reacts differently to a toxic substance when grown in single-cell-type cultures than in mixed-cell type cultures (Eskes et al., 2002; Eskes et al., 2003). Therefore, complex 3D cell culture systems containing the main brain cell types, allowing for maximum cell-to-cell interactions and recapitulating relevant neurodevelopmental processes, are promising tools for the evaluation of the adverse effects of chemicals on the nervous system (NS). The BrainSphere (BS) model is one such complex 3D cell culture system meeting these requirements (Pamies et al., 2017). This model consists of electrophysiologically active neurons, astrocytes, and oligodendrocytes that are able to form compact myelin sheaths around axons. BSs have already been proved to be reliable tools for *in vitro* neurotoxicity testing (Pamies et al., 2018; Zhong et al., 2020; Chesnut et al., 2021; Modafferi et al., 2021; Nunes et al., 2022). They were recently used to decipher the interactions between genes and the environment in developmental neurotoxicity (Modafferi et al., 2021).

As chemical properties, such as lipophilicity, and *in vitro* assay setup properties, such as the level of the serum protein and cell density, can influence the extent to which a chemical accumulates at the target in a cell *in vitro*, an understanding of the *in vitro* distribution kinetics of test chemicals in complex cell models, such as BSs, is essential for comparing chemical potencies and assay sensitivity (Groothuis et al., 2015; Kramer et al., 2015; Honda et al., 2019; Proenca et al., 2021). Traditionally, the nominal effect concentration (EC) has been used to describe *in vitro* neurotoxic potencies. However, the nominal concentration is not a suitable proxy for the freely available concentration of chemicals, especially for lipophilic, volatile, and instable chemicals (Groothuis et al., 2015). Test chemicals differentially evaporate and bind to the microtiter plate plastic and cells (Gulden et al., 2001; Heringa et al., 2004; Jager et al., 2011; Groothuis et al., 2019). Moreover, the cell-associated dose will increase with every repeat dosing of chemicals that significantly accumulate in cells (Wilmes et al., 2013; Kramer et al., 2015). Uncertainties in dose extrapolation are exacerbated in repeat dose toxicity tests, since it is difficult to determine if the increased toxicity observed over time is due to the accumulation of the test chemical in the model system or the “accumulation” of toxic effects (i.e., damage accrual in time). The lack of regulatory acceptance of *in vitro* methods for repeated exposure scenarios may in part be explained by these uncertainties (Mahony et al., 2020).

Here, we assessed the neurotoxicity and distribution kinetics of the very lipophilic drug amiodarone (AMI) in BrainSphere cultures. We used several *in vitro* readouts (targeted transcriptomics, gene expression, and immunohistochemistry), and we calculated different dose metrics (maximum concentration (C_max_) and area under the concentration–time curve (AUC) in the medium and cells) to determine the neurotoxic potency. AMI is a particularly lipophilic basic drug with an estimated log P of 7.64 (Chemaxon, https://www.chemaxon.com), and thus, unlike most drugs, it is likely to accumulate significantly in BrainSpheres upon repeated exposure (Kramer et al., 2015). AMI is an effective antiarrhythmic drug frequently used in clinical practice (Marcus et al., 1981; Hamilton et al., 2020). It inhibits sodium and calcium L-type channels, modulates the potassium outward current, and has antagonistic effects on adrenergic receptors in the heart (Polster and Broekhuysen, 1976; Varro et al., 1996). The long-term use of amiodarone has many side effects, including cardiac, pulmonary, hepatic, and neurological toxicities, the most common being tremor, ataxia, and peripheral neuropathy (Jafari-Fesharaki and Scheinman, 1998; Hindle et al., 2008; Niimi et al., 2019). In addition, some cases of parkinsonism have also been reported (Ishida et al., 2010). However, the mechanisms of neurotoxicity remain unclear, and research in this area is scarce.

## 2 Materials and methods

### 2.1 Cell culture

The hiPSC cell line SBAD3 clone 1 was generated from fibroblasts (Lonza) using Sendai transfection in the StemBANCC project (Morrison et al., 2015). We obtained this cell line through our participation in this project. The cells were cultured in the serum-free mTeSR™ medium (STEMCELL Technologies) in dishes coated with Corning^®^ Matrigel^®^ hESC-Qualified Matrix, LDEV-free (∼18 μg/cm^2^, Corning), at 37°C in 5% CO_2_. The medium was replaced every day. The cells were passaged every 4–5 days using Versene^®^ (Life Technologies). All experiments were carried out following the Good Cell Culture Practice guidelines 2.0 (Pamies et al., 2022). SBAD3 was tested for *mycoplasma* infection using the Mycoplasmacheck service provided by Eurofins Genomics (Ebersberg, Bayern, Germany) before the derivation of NPCs.

The Ad3G2 neuroprogenitor cells (NPC) were generated from the hiPSC SBAD3 clone 1 (passage 22), following the protocol of the “Induction of Neural Stem Cells from Human Pluripotent Stem Cells Using Gibco PSC Neural Induction Medium” (Nunes and Zurich, 2020). Non-confluent cultures of hiPSCs (2.5 × 10^5^ cells/well) in six-well plates (Costar^®^ 3516) were grown in the PSC Neural Induction Medium (NIM, 10% Neural Induction Supplement in Neurobasal^®^ Medium) (Gibco) for 7 days. For expansion, Ad3G2 cells were kept in Geltrex (Gibco)-coated flasks in the Neural Expansion Medium (NEM) containing 45% Neurobasal^®^ Medium (Gibco), 45% Advanced™ DMEM/F-12 Medium (Gibco), and 10% Neural Induction Supplement (Gibco). NPCs were then passaged once per week with StemPro^®^ Accutase^®^ (Gibco) at 70%–90% confluency and reseeded at 9 × 10^4^ cells/cm^2^. The medium was changed every other day. Cultures were maintained at 37°C in 5% CO_2_. Ad3G2 cells were tested for *mycoplasma* infection using the Mycoplasmacheck service by Eurofins Genomics (Ebersberg, Bayern, Germany) twice a year.

BrainSpheres were prepared from the NPC line Ad3G2, as previously described (Pamies et al., 2017). NPCs (passages 10–13) were plated on non-coated six-well plates (2 × 10^6^ cells/well) in 2 mL of the NEM. The medium was gradually replaced with the Neuronal Differentiation Medium (NDM, Neurobasal^®^ Electro Medium (Gibco) supplemented with 2% B-27^®^ Electrophysiology Kit (Gibco), 2 mM L-alanyl-L-glutamine dipeptide (GlutaMAX^TM^, Gibco), 100 U/mL penicillin–100 μg/mL streptomycin (Gibco), 0.01 μg/mL human recombinant glial cell-derived neurotrophic factor (GDNF, PeproTech), and 0.01 μg/mL human/murine/rat recombinant brain-derived neurotrophic factor (BDNF, PeproTech). The medium was replaced three times a week. Cultures were maintained at 37°C in 5% CO_2_, under constant gyratory shaking (86 rpm).

### 2.2 Amiodarone exposure

**FIGURE 1:**
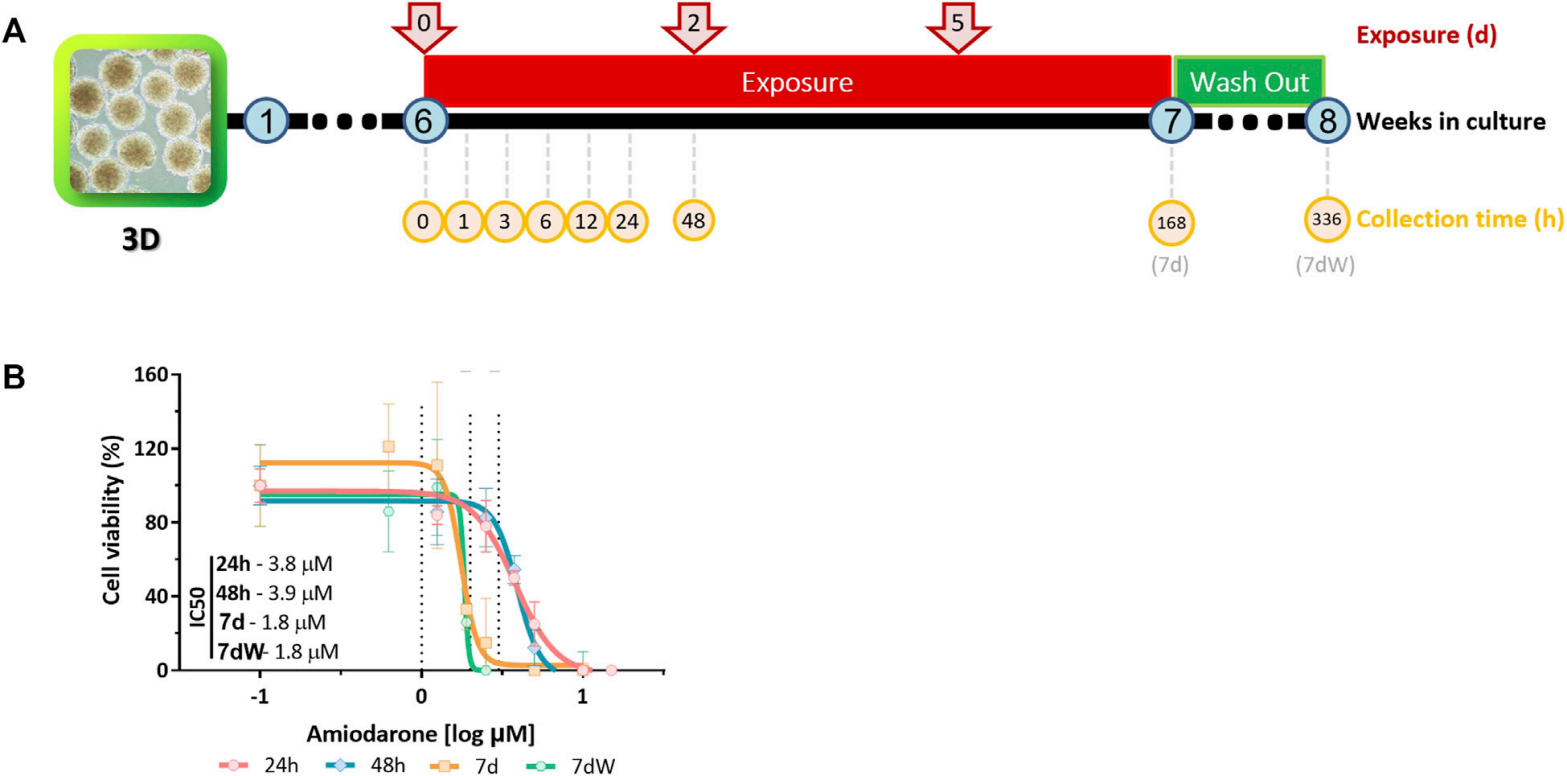
Amiodarone-induced cytotoxicity in BrainSpheres. **(A)** Schematic description of the exposure and sample collection scenarios tested. Spheres were exposed to AMI after 6 weeks of differentiation. The samples were collected (yellow circles) 1, 3, 6, 24, and 48 h after the first exposure (red arrows); after 1 week of repeated exposure (168 h = 7 d); and after a washout period (336 h = 7 dW). **(B)** Nominal concentration–response relationships after one single (24 h and 48 h) or repeated (7 d and 7 dW) exposure to AMI (0–15 μM). The results are expressed as % of control cultures (BS exposed to 0.1% DMSO v/v). Each point is the mean ± SD of 3–9 samples obtained in three independent experiments. A non-linear regression [log(inhibitor) *vs.* response–variable slope (four parameters)] was performed in order to calculate the inhibitory concentration (IC) using Prism^®^.

Amiodarone (purity ≥98%; Sigma-Aldrich, cat. A8423-1G, lot #SLBW3654V) was dissolved in dimethyl sulfoxide (DMSO) (purity ≥99.9%; Sigma-Aldrich, cat. D2650). Stock solutions in DMSO were prepared for each experiment and further diluted 1,000× in the medium to reach final exposure concentrations. Six-week-old BSs were acutely (1–48 h) or repeatedly (7 days, referred to as 7 d or 168 h) exposed to AMI (at each medium change, that is, three times; Figure 1A). The repeated exposure was followed by a week without AMI, called the washout period (referred to as 7 dW or 336 h; Figure 1A). The concentrations of AMI, time points, and numbers of samples per group are specified in the figure caption of Figure 1.

### 2.3 Cytotoxicity assay

Cytotoxicity was measured after 24 h, 48 h, 7 d, and 7 dW of exposure to 0, 0.625, 1.25, 1.9, 2.5, 3.75, 5, 10, and 15 μM AMI (Figure 1A). The exposure medium was replaced by 1 mL of resazurin solution (44 μM), and BSs were incubated for 3 h at 37°C. The fluorescent product was measured at 540/590 nm ex/em with a Synergy plate reader (BioTek). After the subtraction of the background (resazurin solution only), the results were expressed as the % of control cultures (cells exposed to 0.1% of DMSO). A non-linear regression [log(inhibitor) *vs.* response–variable slope (four parameters)] was performed to derive cytotoxic concentration–response relationships at each time point.

### 2.4 Real-time RT–PCR analyses

Upon collection, the samples were washed twice with Dulbecco’s phosphate-buffered saline (DPBS) and dry pellets were kept at −80°C. Total RNA was extracted automatically (QIAcube instrument, Qiagen) using QIAshredder and RNeasy columns (Qiagen). Reverse transcription was performed using 0.5–1 µg of total RNA with the high-capacity cDNA reverse transcription kit (Life Technologies) on the 2720 Thermo Cycler (Applied Biosystems). Semi-quantitative real-time PCR analyses were performed using the 7900HT Fast Real-Time PCR System (Applied Biosystems), with SYBR Green^®^ or TaqMan^®^ (Thermo Fisher) technology in a total volume of 10 µL (Supplementary Tables S1, S2). Each sample was analyzed in triplicate. The thermal conditions were as follows: an initial two-step holding stage at 50°C for 2 min and subsequent denaturation at 95°C for 10 min, followed by 40 cycling stages of denaturation at 95°C for 15 s and annealing/extension at 60°C for 1 min. A final melting curve stage was added when applying SYBR Green chemistry. The ΔΔCt method (Livak and Schmittgen, 2001) was used to calculate the relative mRNA expression. Data were accepted at <40 cycles of amplification. The results are expressed as fold change to DMSO cultures, set at 1. *RLP13A* was used as the reference gene.

### 2.5 Immunohistochemistry

Upon collection, BSs were washed twice with DPBS, fixed for 1 h with 4% paraformaldehyde, and kept in DPBS at 4°C. Fixed BSs were incubated for 2 h in a blocking solution (10% normal goat serum (NGS) (Thermo Fisher) in DPBS with 4% Triton X-100) at 4°C. BSs were then incubated for 48 h at 4°C with primary antibodies (Supplementary Table S3) diluted at a ratio of 1:200 in DPBS, containing 10% NGS and 1% Triton X-100. BSs were washed three times for 5 min in DPBS and incubated for 1 h at room temperature (RT) with the correspondent secondary antibody (Supplementary Table S3) diluted at a ratio of 1:200 in DPBS, containing 10% NGS. BSs were washed three times for 5 min in DPBS, and nuclei were stained with Hoechst 33342 (1:10,000 in PBS, Thermo Fisher) for 5 min. Finally, BSs were mounted on glass slides and coverslips with the ProLong™ Gold Antifade reagent (Thermo Fisher). Z stacks were acquired using a Leica Thunder Imaging System. The images were adjusted and quantified for the “mean gray value” of a z-stack projection for the “maximum intensity” using ImageJ^®^ Fiji.

### 2.6 Statistical analyses

Prism (version 9.01, GraphPad Software) was used for the statistical analysis and graphical representation of the real-time RT–PCR analyses and immunohistochemistry results. Statistical analysis was performed using mixed-effects models with the Geisser–Greenhouse correction followed by Dunnett’s multiple-comparison test. Statistically significant different comparisons are presented in the figures as *p <0.05, **p <0.01, ***p <0.001, or ****p <0.0001. Data are shown as mean ± SD.

### 2.7 *In vitro* distribution kinetics

#### 2.7.1 Sample collection and HPLC analysis

AMI was extracted from the medium, cells, and well-plate plastic and analytically quantified by HPLC-UV. AMI was extracted after 1 h, 3 h, 6 h, 24 h, 48 h, 168 h (7 d), and 336 h (7 dW) of exposure to 1 and 2 µM AMI and after 1 h, 3 h, 24 h, and 48 h of exposure to 3 µM. The medium was collected and centrifugated at 300 rpm for 5 min at 4°C. Then, the supernatant was diluted 1:1 with methanol (purity ≥99.92%; Sigma-Aldrich, catalog no. 900688, EUA). BSs were washed twice with DPBS and transferred to Eppendorf tubes containing 250 μL of methanol. Cell extracts were stored at −80°C. BSs were sonicated to homogenize the sample and centrifugated at 1,200 *g* for 10 min, and the supernatant was collected. To extract AMI from the well-plate plastic, wells were washed with DPBS after BS removal and then incubated with 1 mL of methanol (Sigma) for 2 h at RT using an orbital shaker. AMI was also extracted from the well-plate plastic and medium from exposed *in vitro* systems without cells. All extracts were stored at −20°C. Before HPLC analysis, the extracts were vortexed.

The HPLC system consisted of several modules: two Shimadzu LC-20AD XR liquid chromatograph pumps, a Shimadzu SIL-20A XR autosampler, a Shimadzu CTO-20A column oven, a Shimadzu SPD-20AV UV-VIS detector, an RF-20A XS fluorescence detector, and a Shimadzu CBM-20A Communication Bus Module. A Dr. Maisch GreatSmart RP column (18.5 µm 150 × 2 mm) was used as a stationary phase. Milli-Q water (eluent A) and acetonitrile with 0.1% formic acid (eluent B) were used as a mobile phase with a flow rate of 0.2 mL/min. AMI was trapped in the stationary phase with 5% eluent B. Then, eluent B rose to 95% in 0.5 min and was maintained for the remainder of the 7-minute run, after which it decreased back to 5%. Amiodarone peaks were detected after 6.3 min at 242 nm UV wavelengths. The mass of AMI was quantified using calibration standards prepared in the respective matrix.

#### 2.7.2 *In silico* modeling of AMI *in vitro* distribution kinetics

**FIGURE 2:**
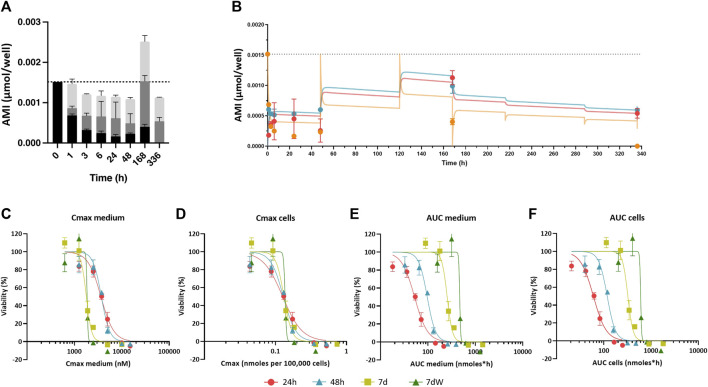
Amiodarone distribution kinetic: *in vitro* experimental values, *in silico* prediction, and concentration–effect relationships based on various dose metrics. **(A)** Distribution of the amount of AMI in the medium (black bars), cell lysates (gray bars), and plastic binding (light gray bars) after acute (1–48 h) and repeated treatment (168 h = 7 d and 336 h = 7 dW) to 1 µM AMI. The results are reported as mean ± SD of three biological replicates. **(B)** Kinetic profiles of AMI experimentally measured in cell lysates (red circles), in the medium (orange circles), and in the plastic bound fraction (blue circles) of BSs exposed to AMI 1 µM. Each value is the mean (± SD) of three replicates. Predicted curves are superimposed on experimentally measured values, cells (red line), medium (orange line), and plastic binding (blue line). Dotted line represents the quantification of AMI in the medium at time = 0. **(C–F)** Concentration–effect relationships after single (24 h, red dots, and 48 h, turquoise triangles) and repeated (7 d, olive squares, and 7 dW, green triangles) dosing to 1–15 μM AMI in the medium were calculated using different dose metrics: **(C)** C_max_ in the medium, **(D)** C_max_ in the cells, **(E)** AUC of the amount in the medium, and **(F)** AUC of the amount in the cells. All results are expressed as % of control cultures (DMSO). Each point is the mean ± SD of 3–9 samples obtained in three independent experiments.

To model the distribution of AMI in BS systems after repeated exposure, three model compartments were defined: medium, cells, and plastic (Supplementary Figure S1A). For cell-free assays, two model compartments were defined: plastic and medium. The change in AMI mass in each compartment was defined using rate constants in and out of each compartment (e.g., ka_plastic_ and kd_plastic_ denoting sorption and desorption rate constants in plastic). The profile of AMI distribution in the *in vitro* system (in the presence and absence of cells) in time suggests that there is a slow and irreversible loss of AMI from the system (Figure 2A; Supplementary Figure S3, S5). This process was herein considered to be the abiotic degradation (K_deg_) of the medium.

To develop the *in vitro* kinetics model, only data of non-cytotoxic concentrations were used. All the rate constants were fit by minimizing the error between the predictions of the AMI amount in the different compartments after 1 and 2 µM acute exposure and 1 µM repeated exposure and the experimental data (Supplementary Figure S1B). The error between prediction and experimental values were normalized not only to the respective experimental value but also to the time point, to make sure the prediction errors for the different nominal concentrations, compartments, and time points were balanced. Plots of the different residuals are presented in Supplementary Figure S2. Minimization was obtained through the Broyden–Fletcher–Goldfarb–Shanno (BFGS) algorithm implemented using the optim function in R. The differential equations used to simulate the wells with cells are illustrated in Supplementary Figure S1C. The model was then used to calculate the different dose metrics for dose–response modeling. To calculate the maximum concentration (C_max_) and area under the curve (AUC) in cells, we assumed the cell number after exposure was the same as that at the time of seeding (two million cells/well). The model was then used to simulate amounts of AMI in the medium, cells, and plastic in *in vitro* systems exposed to cytotoxic concentrations (2 µM of repeated exposure and 3 µM of acute exposure). The model was written and simulated in R (version 3.63), using the RStudio interface (https://github.com/Proen1/Brainspheres_Amiodarone).

### 2.8 TempO-Seq analysis

#### 2.8.1 TempO-Seq sample collection

At the end of exposure to AMI, the cells were collected and lysed with 1× TempO-Seq Lysis Buffer (BioClavis) in a ratio of 0.25 to 2 million cells/mL. Lysates were frozen at −80°C and shipped to BioClavis Technologies Ltd. (Glasgow) on dry ice, where the TempO-Seq assay using the EU-ToxRisk v2.1 panel (3,565 probes representing 3,257 genes) was conducted with standard attenuators. The service also included primary processing to derive gene-annotated raw read counts and quality control, following a previously described procedure (Limonciel et al., 2018; Mav et al., 2018). Each sample FASTQ file was aligned against the TempO-Seq transcriptome using the Bowtie aligner (Li and Durbin, 2009). The output of this analysis generated a table of counts per gene per sample (Supplementary Material raw data and metadata).

#### 2.8.2 Differential expression analysis

Differential gene expression analyses were performed in R using DESeq2 v1.32.0 built-in functions (Love et al., 2014). Unsupervised clustering of the samples was performed after variance-stabilizing transformation (VST) using standard DESeq2 suggested settings to visualize the variance within and between treatment and control groups. To normalize for sequencing depth and RNA composition, DESeq2 uses the median of ratios method. To identify differentially expressed genes (DEGs) or probes, DESeq2 uses by default the Wald test. The probes indicating differentially expressed genes were selected based on their multiple testing corrected adjusted *p*-value (<0.05).

#### 2.8.3 Gene Ontology analysis

Overrepresentation (or enrichment) analysis was performed on the differentially expressed probes using the *enricher* function in clusterProfiler v4.0 (Yu et al., 2012) from Bioconductor v4.1.0 and the GO biological process gene sets from MSigDB at Broad Institute v7 (Subramanian et al., 2005; Liberzon et al., 2011). The lipid metabolism linked gene heatmap was generated using the heatmap package in R.

## 3 Results

### 3.1 Bioaccumulation of amiodarone leads to increased cytotoxicity

Cytotoxicity was assessed after 24 h, 48 h, 168 h (7 d), and 336 h (which includes a 7-day washout period, 7 dW) of exposure to 0 and 1–15 µM AMI using the resazurin assay (Figure 1A). Expressed as nominal median inhibitory concentrations (IC50), AMI’s cytotoxic potency increased with the exposure time (Figure 1B). There was no significant difference in IC50 at the end of 7 days of exposure to AMI (7 d) and after the washout period (7 dW). The slopes of the curves are steep, particularly after 7 days of treatment (Figure 1B, 7 d and 7 dW).

The distribution kinetics of AMI in our *in vitro* system was assessed to explain the observed time-dependent cytotoxicity. After 0 h, 1 h, 3 h, 6 h, 24 h, 48 h, 168 h (7 d), and 336 h (7 dW) of exposure to 1 and 2 µM AMI and after 0 h, 1 h, 3 h, 6 h, 24 h, and 48 h of exposure to 3 μM, the amount of AMI in the cells, medium, and well-plate plastic was analytically determined (Figure 2; Supplementary Figure S3). Repeated exposure was not conducted with the highest concentration due to its cytotoxicity. On average, 100% of AMI was recovered from the system after 1–24 h of exposure, but this value decreased to 85% after 48 h exposure. The loss of AMI at 48 h was not considered to be due to metabolism, as the main oxidative metabolite of AMI, mono-N-desethylamiodarone (MDEA), was not detected in the cells or medium extracts (data not shown).

AMI showed slow cell uptake kinetics. At 1 µM AMI, 12% of the mass accumulated in cells after 1 h, whereas 45% was found in cells after 24 h (dark gray bars in Figure 2A and red dots in Figure 2B; Supplementary Figure S4 is added to better appreciate the experimental values at short time points). AMI peaked in cells after 7 days (168 h) and then decreased during the washout period (336 h). Because AMI is retained in cells and plastic when the exposure medium is refreshed, the total recovery of AMI after 168 h of exposure is more than the mass added to the medium and AMI is still detected in cells and plastic after the washout period (dark and light gray bars in Figure 2A; red and blue dots in Figure 2B; 336 h). Binding to plastic was found to be quicker than sorption in cells (light gray bars in Figure 2A ; blue circles in Figure 2B), reaching its maximum after 7 days, followed by slow desorption during the washout phase. The amounts in plastic and cells measured after 2 and 3 µM AMI exposures (Supplementary Figure S3A–C) showed the same pattern as the 1 µM AMI exposure, despite the fact that these higher AMI concentrations resulted in cytotoxicity after 7 days of exposure.

Altogether, these data show an increased cytotoxicity of AMI with repeated exposure and suggest that it is due to its bioaccumulation in cells. The *in silico* compartmental model of *in vitro* kinetics can predict this accumulation of AMI in the medium and cells within 2.5-fold of experimental values, including exposures where cytotoxicity was observed. The compartmental model is based on the model described in the work of Pomponio et al. (2015a; 2015b). Here, three compartments representing the concentrations of amiodarone in the medium, cells, and plastic are used and the rate constants in and out of BSs and on and off plastic were fit using the first measured concentrations in medium, plastic, and cells within the first 24 h after exposure to non-cytotoxic 1 µM amiodarone. Concentration–time profiles in the medium and plastic of systems free of cells (Supplementary Figure S5) were used to correct for assumed abiotic degradation of AMI. The best-fit values for sorption and desorption rate constants into cells and plastic are given in Supplementary Figure S2C. The model was able to predict the measured concentrations in each compartment at the higher exposure levels and repeated exposures, suggesting that the model accurately captures the accumulation processes of amiodarone in BS systems. This illustrates how compartmental modeling can be used to predict accumulation at repeated doses and different dose levels with just a few analytical measurements of the cell, plastic, and medium concentrations of test chemicals at the start of exposure, as has been shown in previous studies with hepatocytes and rodent brain cell cultures (Pomponio et al., 2015a; Pomponio et al., 2015b; Kramer et al., 2015).

### 3.2 C_max_ in cells is a setup-independent dose metric for dose response modeling

The compartmental model was applied to simulate the distribution and accumulation of amiodarone in the different *in vitro* compartments for all nominal test concentrations and exposure scenarios. While it is common to assume that the free concentration in the medium is equivalent to the free concentration in cells, the slow uptake of amiodarone in the cells in BrainSpheres indicates that this assumption is unlikely to hold. Moreover, calculating the free amount in the medium requires full knowledge of the composition of the cell culture medium, which is not the case for supplements such as B27. A highly lipophilic chemical, such as AMI, requires advanced binding affinity determination techniques, such as solid-phase microextraction, for determining free fractions in the medium. All things considered, nominal concentrations were used instead of determining the free concentrations of AMI in the medium.

This simulation allowed us to calculate the C_max_ in the medium and cells and the AUC of the amount in medium and cells. Figure 2C–F shows how plotting the C_max_ in cells against viability causes concentration–effect curves for each exposure scenario to cluster together. IC50 values as C_max_ in the cells ranged from 0.1559 (7 d) to 0.1626 (7 dW) nmoles per 100,000 cells (Table 1). The concentration–effect curves using the AUC medium and AUC cells as dose metric clustered less closely together than the curves using C_max_ in the medium as the dose metric. When using AUC in the medium as a dose metric, 24-h single exposure showed the lowest IC50 value (58.0 nmol·h) and 7 dW showed the highest IC50 value (410.2 nmol·h) (Table 1). When using the AUC in cells as a dose metric, 24-h single exposure resulted in the lowest IC50 value (73.9 nmol·h) and 7 dW exposures gave the highest IC50 value (604.1 nmol·h) (Table 1).

**TABLE 1:** IC50 values for the different dose metrics calculated through [inhibitor] *vs.* normalized response–variable slope least-squares fit regression using GraphPad Prism^®^.

	C_max_ medium (µM)	C_max_ cells (nmoles/100,000 cells)	AUC medium (nmoles·h)	AUC cells (nmoles·h)
	IC50 (95% CI)	IC50 (95% CI)	IC50 (95% CI)	IC50 (95% CI)
24 h	3.63 (3.03–4.23)	0.127 (0.106–0.147)	52.5 (43.8–61.1)	60.1 (50.2–70.1)
48 h	3.70 (3.15–4.21)	0.129 (0.110–0.147)	97.0 (82.4–110.2)	117.9 (100.1–133.8)
7 d	1.79 (1.61–1.97)	0.132 (0.119–0.145)	255.6 (230.0–281.1)	322.9 (290.5–355.2)
7 dW	1.85^a^	0.137^a^	469.0^a^	602.8^a^

CI, confidence interval; ^**a**^95% CI cannot be calculated due to ambiguous curve fitting.

### 3.3 Amiodarone has a deleterious effect on neurons and astrocytes

**FIGURE 3:**
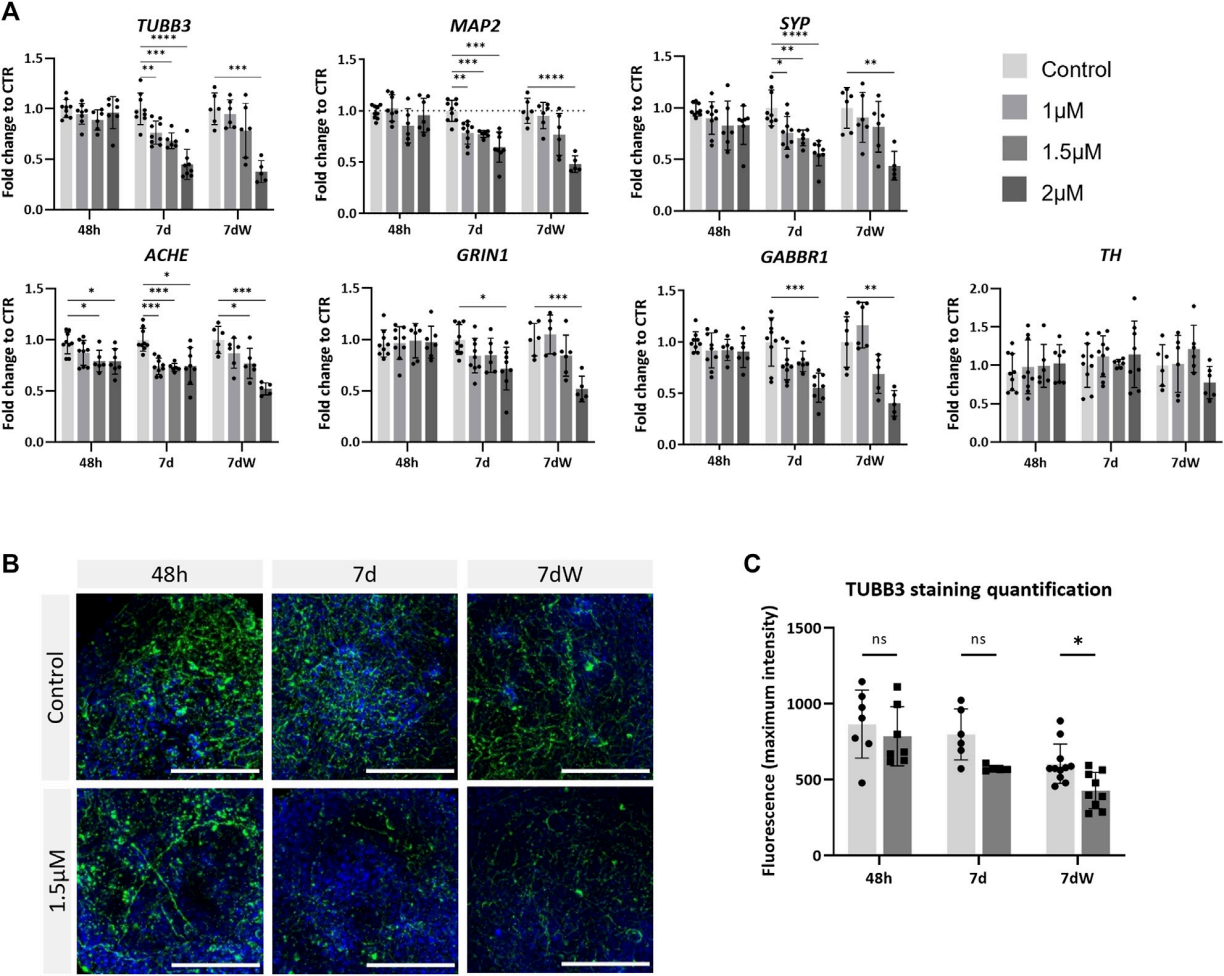
Neurons are affected by amiodarone. **(A)** Relative gene expression of neuronal (*TUBB3* and *MAP2*) markers, pre-synaptic (*SYP*) markers, catecholamine-containing neuron (*TH*) markers, acetylcholinesterase (*ACHE*), glutamate receptor ionotropic NMDA type subunit 1 (*GRIN1*), and gamma-aminobutyric acid type B receptor subunit 1 (*GABBR1*). Data are expressed as fold change to control. Each value is the mean (± SD) of 3–9 samples coming from three independent experiments. **(B)** Immunostaining for TBB3 (green) was carried out in the BS control and treated with 1.5 µM AMI at 48 h, 7 d, and 7 dW; nuclei are stained with Hoechst (blue). Scale bars indicate 50 μm. **(C)** Quantification of the maximum intensity of TBB3 immunostaining. Each value is the mean (± SD) of 3–5 spheres. *p <0.05, **p <0.01, ***p <0.001, and ****p <0.0001.

To assess neurotoxicity, BSs were treated with various concentrations of AMI (1, 1.5, and 2 µM) and were collected after an acute (48 h; all concentrations tested were <IC10 for cytotoxicity) or repeated (1 week; 1 µM <IC10; 1.5 µM <IC20; 2 µM >IC50) exposure. After 1 week of exposure, AMI induced a concentration-dependent significant decrease in the mRNA levels of the neuronal markers tubulin beta-3 chain (*TUBB3*), *MAP2*, and *SYP* (Figure 3A). Markers of neuronal subtypes showed that AMI also significantly decreased the expression of *ACHE*, which is indicative of the presence of cholinergic neurons, already after 48 h of treatment, whereas the level of the *TH* mRNA, present in catecholaminergic neurons, was not modified at any time point. The expression of the NMDA receptor *GRIN1* and of the GABA receptors *GABBR1* was reduced after 7 days of exposure. The results after the washout period (7 dW) were very similar to those observed at the end of the 7-day treatment, 7 d with only a slightly stronger decrease observed after the highest concentration (2 µM). Immunostaining (Figure 3B) showed a decreased network of fibers stained for TBB3 after 1 week of exposure to AMI and after the washout period. Image quantification confirmed this decrease, although it was statistically significant only after the washout period (Figure 3C).

**FIGURE 4:**
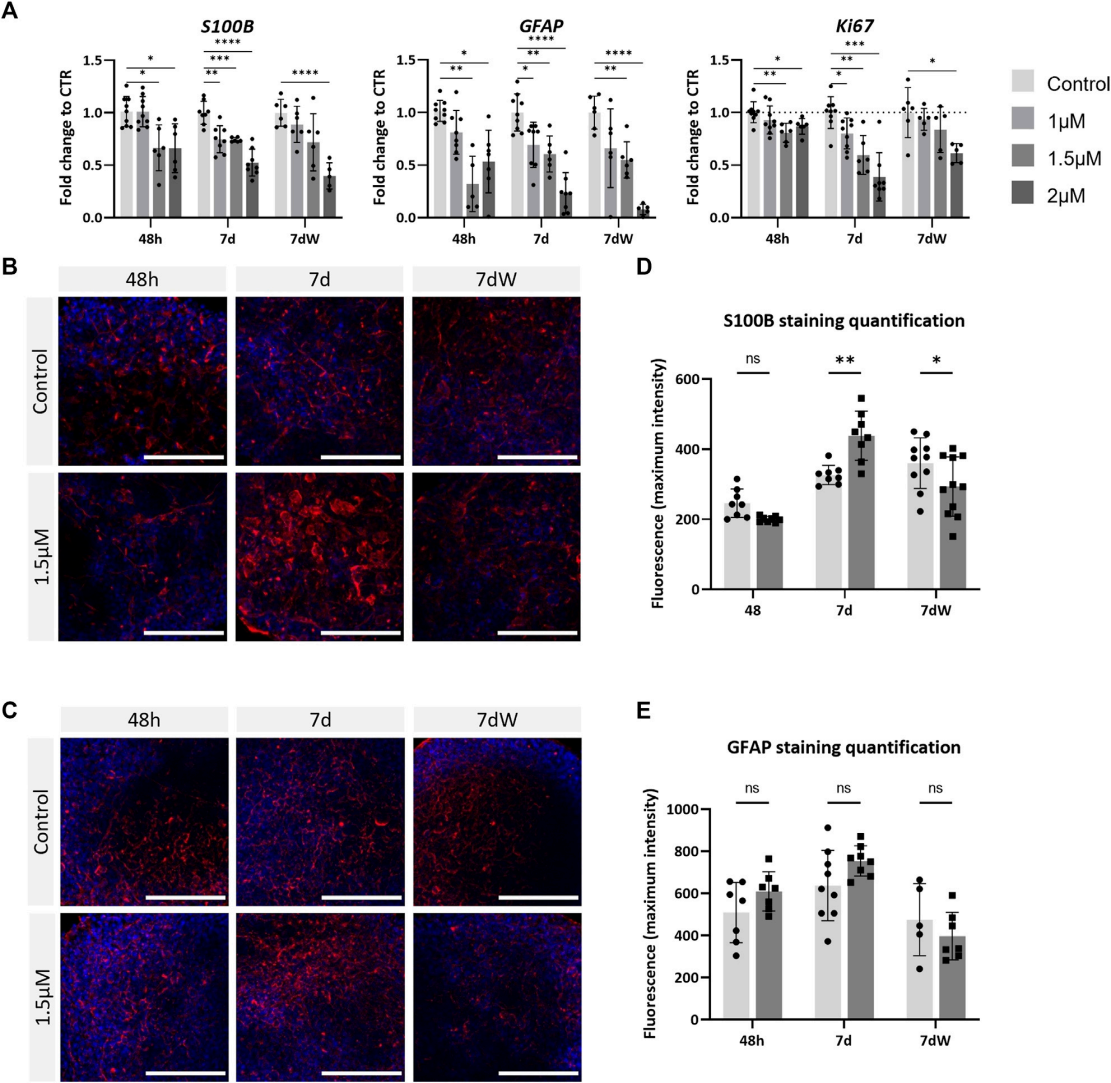
Astrocytes are strongly affected by amiodarone. **(A)** Relative gene expression of astrocytic markers (*GFAP* and *S100B*) and *Ki67*. Data are expressed as fold change to the control. Each value is the mean (± SD) of 3–9 samples coming from three independent experiments. **(B)** Immunostaining for S100B (red) and **(C)** GFAP (red) is carried out in the BS control and treated with AMI; nuclei are stained with Hoechst (blue). Scale bars indicate 50 μm. **(D)** Quantification of the maximum intensity of S100B immunostaining and **(E)** GFAP. Each value is the mean (± SD) of 3–5 spheres. *p <0.05, **p <0.01, ***p <0.001, and ****p <0.0001.

AMI significantly decreased the mRNA levels of *S100B* and *GFAP* in a concentration-dependent way, already after 48 h of exposure (Figure 4A), with a slightly stronger effect on *GFAP*. The results were very similar after repeated exposure (7 d) and the washout period (7 dW). Contrarily, increased immunostaining for *S100B* was observed after AMI repeated exposure (7 d) (Figures 4B,D). At that time point, astrocytic processes appeared thicker than those in the control cultures (Figure 4B, 7 d), and quantification showed a significant increase (Figure 4D). These changes in immunolabeling were not present anymore after the washout period (7 dW), and a significant decrease was quantified. No change was observed in GFAP immunostaining (Figures 4C,E). *Ki67*, a marker of proliferation, was slightly but significantly downregulated 48 h after the first treatment, more strongly after 1 week, and a slight recovery in the mRNA level was observed after the washout period (Figure 4A). Finally, AMI did not produce any change in the expression of the oligodendrocyte transcription factor 2 (*Olig2*) and myelin basic protein (*MBP*) gene (Supplementary Figure S6A), nor in the immunostaining for the O4 protein (Supplementary Figure S6B, C).

These results suggest that brain cells are susceptible to AMI, with astrocytes possibly being affected earlier and more strongly than neurons.

### 3.4 TempO-Seq analysis highlights lipid metabolism as an important target of AMI action

**FIGURE 5:**
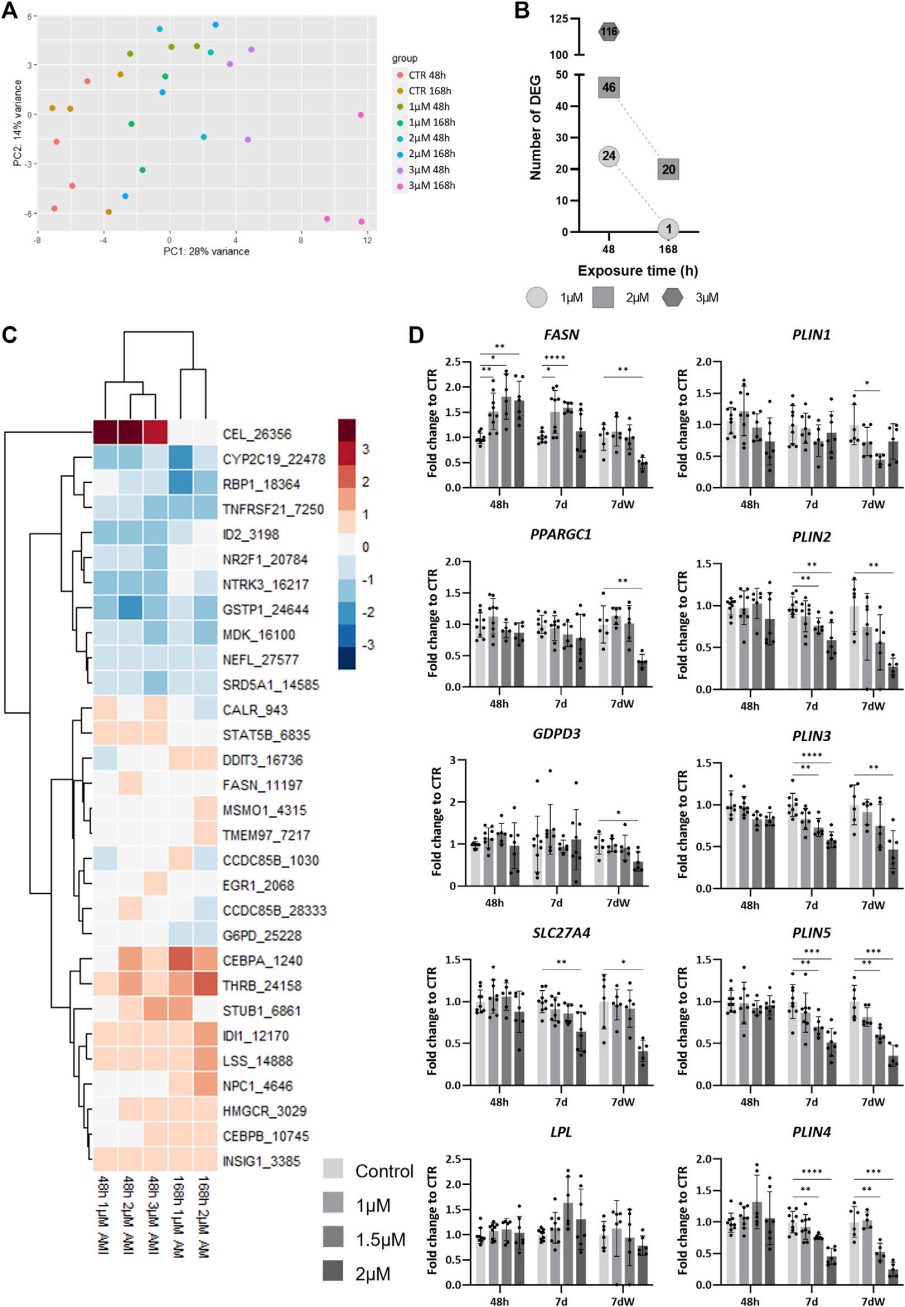
TempO-Seq analysis of BrainSpheres exposed to amiodarone. **(A)** PCA of all control- and AMI-treated samples. Each dot represents a sample, and each color represents a group (control or treated). **(B)** Number of DEGs per condition. **(C)** Heatmap of the log_2_ fold changes compared to control of genes linked to lipid biological processes, in all treated groups. Red: upregulation, blue: downregulation; **(D)** relative gene expression of markers of fatty acid transport (*SLC27A4*), mitochondrial biogenesis (*PPARGC1*), *de novo* lipogenesis (*FASN*), degradation of phospholipids (*GDPD3*), lipid hydrolysis (*LPL*), and the formation of lipid droplets (*PLIN1-5*). Data are reported as fold change to the control, and each value is the mean (± SD) of 3–9 samples coming from three independent experiments. *p <0.05, **p <0.01, ***p <0.001, and ****p <0.0001.

To evaluate the alteration of the cellular program in response to AMI exposure, we performed TempO-Seq analysis for a set of genes (3,257) selected for their representation in toxicity-related pathways. The unsupervised PCA of the samples exposed to AMI (1, 2, and 3 µM) for 48 h or 7 d showed that the samples cluster along the *x*-axis (PC1) in accordance with the concentration of AMI they were exposed to (Figure 5A). After differential expression analysis (using DESeq2), we detected 24 differentially expressed genes (DEGs) after 48 h of acute exposure to AMI 1 μM, 46 DEGs for 2 μM, and 116 for 3 µM (Figure 5B). Repeated exposure showed 1 and 20 DEGs after exposure to AMI 1 µM and 2 μM, respectively, which is less than that after acute exposure. The highest concentration (3 µM) was not used for repeated exposure, due to its very high cytotoxicity.

Gene set overrepresentation analysis was performed based on hallmark gene sets from MSigDB at the Broad Institute using the enricher function from the clusterProfiler R package. The retrieved biological processes (BPs) were manually grouped into categories. The three categories with the highest numbers of associated BPs were lipid metabolism, neural function, and differentiation and development (Supplementary Table S4). BPs associated with neural function were only detected after 48 h of exposure to 3 μM, whereas BPs associated with differentiation and development were also detected after 2 µM (48 h and 7 d), and BPs associated with lipid metabolism were found in all conditions, except after repeated exposure (7 d) to 1 µM (Supplementary Table S4).

### 3.5 Amiodarone disrupts lipid metabolism

A total of 20 different BPs associated to lipid metabolism were affected by AMI (Supplementary Table S5). A rapid and transient deregulation of the genes associated to these BPs was observed. Indeed, more DEGs are detected after 48 h than after 7 days (Figure 5B). Some of these BPs present a concentration-dependent increase of the number of DEGs after 48 h of exposure (Supplementary Table S5). Among them, we have the following: “regulation of lipid metabolic process,” “regulation of steroid metabolic process”, “steroid metabolic process,” and “sterol metabolic process”.

A heatmap shows the list of DEGs associated with lipid metabolism BPs extracted from the different conditions (Figure 5C). The expression of two genes is modified only at 48 h; carboxyl ester lipase (*CEL*) is strongly upregulated (log_2_ fold change (LFC) >4), whereas nuclear receptor subfamily 2 group F member 1 (*NR2F1*) is downregulated (LFC <−0.4). The differential expression of some genes was clearly more affected after 1 week of repeated exposure than after 48 h, such as DNA damage-inducible transcript 3 (*DDIT3*), methylsterol monooxygenase 1 (*MSMO1*), transmembrane protein 97 (*TMEM97*), CCAAT enhancer-binding protein alpha (*CEBPA*), thyroid hormone receptor beta (*THRB*), isopentenyl-diphosphate delta isomerase 1 (*IDI1*), lanosterol synthase (*LSS*), and Niemann–Pick type C disease intracellular cholesterol transporter 1 (*NPC1*).

The quantification by real-time RT–PCR showed a concentration-dependent increase in fatty acid synthase (*FASN*) expression after 48 h and 7 days (Figure 5D), whereas after 7 dW, the *FASN* expression went back to control levels, except for a significant decrease at 2 μM, a concentration above the IC50 value for cytotoxicity, i.e., a clearly cytotoxic concentration (Figure 1B). The mRNA levels of peroxisome proliferator-activated receptor gamma coactivator 1 alpha (*PPARGC1*) and glycerophosphodiester phosphodiesterase domain-containing 3 (*GDPD3*) were significantly reduced at 2 µM after the washout period, whereas solute carrier family 27 member 4 (*SLC27A4*) was also decreased at the end of the 7-day repeated exposure period. Lipoprotein lipase (*LPL*) expression was not statistically significantly changed after AMI exposure. Finally, the expression of perilipin (*PLIN*) 2–5 was strongly and significantly diminished after 7 days and 7 dW, whereas *PLIN1* showed only slight changes after the washout period.

Altogether, these data indicate that profound changes in different lipid metabolism-related processes occur in BSs exposed to AMI.

## 4 Discussion

AMI side effects include neurological prejudice. Here, we took advantage of the hiPSC-derived 3D BrainSphere model to integrate distribution kinetics and toxicodynamics to assess its neurotoxic effects *in vitro* in human brain cells. IC50 values for cytotoxicity decreased with increasing exposure time when nominal medium concentrations were used to produce concentration–response relationships. By expressing IC50 values using cell-associated concentrations, the apparent increase in toxicity is explained by the accumulation of the chemical in cells after repeated dosing, illustrating the importance of accounting for *in vitro* distribution kinetics when comparing assay sensitivities, ranking potencies, and QIVIVE (Groothuis et al., 2015; Kramer et al., 2015). The compartmental model in this study, like its predecessors (Wilmes et al., 2013; Pomponio et al., 2015a; Pomponio et al., 2015b), is particularly useful to predict cell-associated concentrations in time and interpret altered *in vitro* potencies upon repeated exposure and at different dose levels in complex *in vitro* models. Currently, mechanistic *in vitro* distribution models using quantitative structure–activity relationships (QSARs) to predict cell-associated concentrations, as reviewed in the work of Proenca et al. (2021), are not yet applicable in complex 3D *in vitro* models with dynamic processes including abiotic and metabolic degradation. Albeit difficult, given the particular challenges associated with predicting permeability and degradation rates from the chemical structure, the next step would be to develop and validate such a mechanistic model for 3D cultures using analytical measurements of cell-associated concentrations of a suite of chemicals and assays after repeat dosing. The compartmental model in this study, although only applicable to the assay setup in this study and amiodarone, was able to capture the changes in the concentration of amiodarone in cells in time within 2.5-fold of the experimental data. To deem if this uncertainty in the prediction of *in vitro* distribution kinetics is acceptable for deriving a point of departure (POD) for QIVIVE, it should be compared to the uncertainty of using the nominal concentration as POD.

The cytotoxicity is the same 24 h and 48 h after a single exposure, and no progression of cell death or proliferation occurred after the removal of AMI, as seen by the similarity of IC50 values after 7 days of exposure and 7 dW. A decrease in the gene expression of the proliferation marker Ki67 after the washout period confirmed the absence of proliferation. These results suggest that the strongest effects observed after repeated dosing is driven by AMI accumulation in cells rather than by the increase in exposure time. This also explains why concentration–effect relationships based on the C_max_ in cells, a threshold concentration rather than the cumulative concentration (AUC), cluster. The IC50 value based on C_max_ in cells suggests that AMI is a baseline toxicant, i.e., it non-specifically disturbs the cell membrane by being present in the membrane (Wezel and Opperhuizen, 1995; Escher et al., 2020), in this cytotoxicity assay (Escher et al., 2011).

The prediction of the dose range for adverse neurological effects of AMI in humans was previously performed by QIVIVE (Algharably et al., 2021), from the results of choline acetyltransferase inhibition, used as a marker of neurotoxicity and obtained with a 3D rat brain cell model (Pomponio et al., 2015b). C_max_ and AUC in cells measured on the last day of 14-day repeated exposure to 1.25 µM of AMI were used. Reverse dosimetry with both dose metrics resulted in very similar external doses. However, AUC represented only the last day and not the entire exposure. In light of the results obtained using 3D human BSs, C_max_ in cells appeared to be the dose metric most independent from the exposure scenario, when considering cytotoxicity. Therefore, C_max_ in cells may be a more robust dose metric than the AUC in cells to use in AMI concentration-response modeling and as a point of departure for QIVIVE, when using *in vitro* toxicity data from the current study.

In the present study, a loss of neurons is suggested by decreased *TUBB3* and *MAP2* gene expression and TBB3 protein level and the absence of recovery after AMI removal. In particular, cholinergic neurons seemed to be more sensitive to AMI than the catecholaminergic ones. These deleterious effects on neurons are in line with previous studies (Turovaya et al., 2005; Chang et al., 2017). Astrocytes seemed to be more affected than neurons and also at an earlier time point. The gene expression of *S100B* and *GFAP* was strongly and rapidly reduced after exposure to AMI, and mRNA levels did not recover after 1 week of washout, suggesting a loss of astrocytes. However, a transient reactivity of the remaining astrocytes is suggested by the modification of S100B immunolabeling observed after 1 week of repeated exposure but not after the washout period. This is in contrast to the slightly higher sensitivity of neurons, observed on gene expression, reported for rat mixed brain cell cultures (Pomponio et al., 2015b). The apparent higher sensitivity of human *vs.* rat astrocytes *in vitro* may be related to their respective state of maturation, human BSs being probably less mature than rat 3D cultures, given the differences in the duration of the developmental processes between the two species. Finally, due to the relationships between the various brain cell types, we can hypothesize that the absence of microglial cells in BSs renders astrocytes more vulnerable to AMI.

To quantitatively assess differences in sensitivity to AMI between *in vitro* models, variations in setups have to be accounted for (Proenca et al., 2021). Differential binding to various cell culture containers would cause concentration–time profiles at cellular targets to vary, despite similar nominal concentrations being added. In previous studies, the mass of AMI in models of human hepatocytes and of mice brain cells (Pomponio et al., 2015a; Pomponio et al., 2015b) decreased after single exposure, similarly to the depletion observed in human BSs, especially after 48 h. For both *in vitro* systems, this was partly attributed to the biotransformation of AMI into MDEA. It cannot be the case for BSs, since no MDEA was found and the same degradation rate constants were estimated with and without cells. Furthermore, CYP3A4, mostly responsible for the formation of MDEA, was neither found in control BSs nor after exposure to AMI (data not shown), in line with its reported absence from the developmental human brain (Yang et al., 1994; Zahno et al., 2011). All these data point toward an absence of AMI metabolism in the BS model. The ratio of the exposed plastic surface area to exposure medium in the different cell culture systems is the same, yet the amount of AMI found in plastic after 24 h of exposure was higher in the BS model. This suggests that higher nominal concentrations, higher cell densities, the presence of extracellular matrix, and 2% serum in the exposure medium limited the binding of AMI to plastic in the mice brain and human liver models.

Whereas cellular accumulation in BS reached 32%–44% of the total added AMI dose after 24 h, 2D mouse brain cells accumulated 60%, and the 3D rat aggregating brain cell model accumulated the total dose (100%) (Pomponio et al., 2015b). Since AMI has not yet been described as a substrate of any specific membrane transporter, differences in cell accumulation may be attributed to variations in the lipid content and lysosome numbers between cell systems. This suggests that small variations in the cell density, ratio of the exposed surface plastic area to exposure medium, and frequency of exposure medium replacement may significantly affect the maximum concentration of AMI at the cellular target. The compartmental model developed and parametrized in this study allowed us to predict the change in time-dependent cell-associated concentrations in BSs for different exposure scenarios. These predictions point to amiodarone accumulating in the cells and, thus, decreasing IC50 values with repeated exposures. In the work of Pomponio et al. (2015b), the rodent brain cells already accumulated amiodarone more extensively at 24 h, and additionally, they were repeatedly exposed for 14 days instead of the 7 days used in this study. Nevertheless, this increased intracellular exposure did not lead to increased toxicity in the rodent model, as discussed above. This amounts to the evidence of the human model being intrinsically more sensitive to AMI exposure. However, for an accurate comparison of the sensitivities, the number of cells in both rodent and human *in vitro* models should be more accurately determined in future experiments.

Numerous biological processes related to the lipid and cholesterol metabolism were found in this study after the gene set overrepresentation analysis of TempO-Seq data. In particular, the upregulation of the genes *MSMO1*, *IDI1*, *LSS*, and *HMGCR* suggested enhanced synthesis of cholesterol, and the upregulation of *FASN* suggested an increased synthesis of fatty acids that may in turn induce phospholipidosis (Sawada et al., 2005; Antherieu et al., 2011). This would be in agreement with the reported induction of phospholipidosis by AMI, in various cell types, such as macrophages, alveolar epithelial cells, and hepatocytes (Lewis et al., 1990; Nonoyama and Fukuda, 2008; Kapatou et al., 2010; Ohlinger et al., 2020). However, this upregulation of lipogenic genes was not accompanied by the upregulation of *PLIN*s that are essential for lipid droplet building and, therefore, lipid storage. These results suggest that the newly produced lipids could be not stored in droplets, but were rather stored in another cellular structure or immediately consumed. However, we have not measured the levels of PLIN proteins. Astrocytes produce lipids more efficiently than neurons, and it has recently been shown that astrocyte lipid metabolism is critical for the development and function of synapses in mice (van Deijk et al., 2017). It may be hypothesized that the highest toxicity observed in astrocytes derives from their higher ability to oxidize fatty acids, as compared to neurons, that may generate harmful products (for review, Schonfeld and Reiser, 2021). Contrary to the other cell types present in BrainSpheres, oligodendrocytes do not seem to be affected by AMI, as seen by the absence of effects on transcriptomic markers and O4 immunostaining, although this drug has been shown to induce demyelination in the peripheral nervous system (Pulipaka et al., 2002; Niimi et al., 2016; Niimi et al., 2019). Further investigations are needed to determine the exact status of oligodendrocytes and of the myelin sheath after the exposure of BSs to AMI.

In conclusion, when correcting for differences in *in vitro* distribution kinetics, our study indicates that human brain cells are intrinsically more sensitive to AMI exposure than rodent brain cell cultures. This study illustrates the benefit of assessing the *in vitro* distribution kinetics of test chemicals to explain variations in effect concentrations between *in vitro* assays differing in setup. The integration of distribution kinetics and toxicodynamics also allowed us to show that the observed time- and concentration-dependent increase in the neurotoxic effects of amiodarone is coupled with its cellular accumulation. Furthermore, our study provides, for the first time, the evidence that AMI induces lipid metabolism perturbation in human brain cells that might be associated with deleterious effects on astrocytes. We believe that *in vitro* determination of neurotoxicity using human-based complex models, such as human iPSC-derived BrainSpheres, coupled with *in vitro* distribution kinetics, is key to refining chemical risk assessment for human health.

## Data Availability

The original contributions presented in the study are included in the article/Supplementary Material, further inquiries can be directed to the corresponding author.
