# Supplementary material for: Integrating distribution kinetics and toxicodynamics to assess repeat dose neurotoxicity in vitro using human BrainSpheres: a case study on amiodarone
Source: Front Pharmacol. 2023 Sep 6;14:1248882. doi: 10.3389/fphar.2023.1248882 (PMC10512064; doi:10.3389/fphar.2023.1248882)

## Slide 1
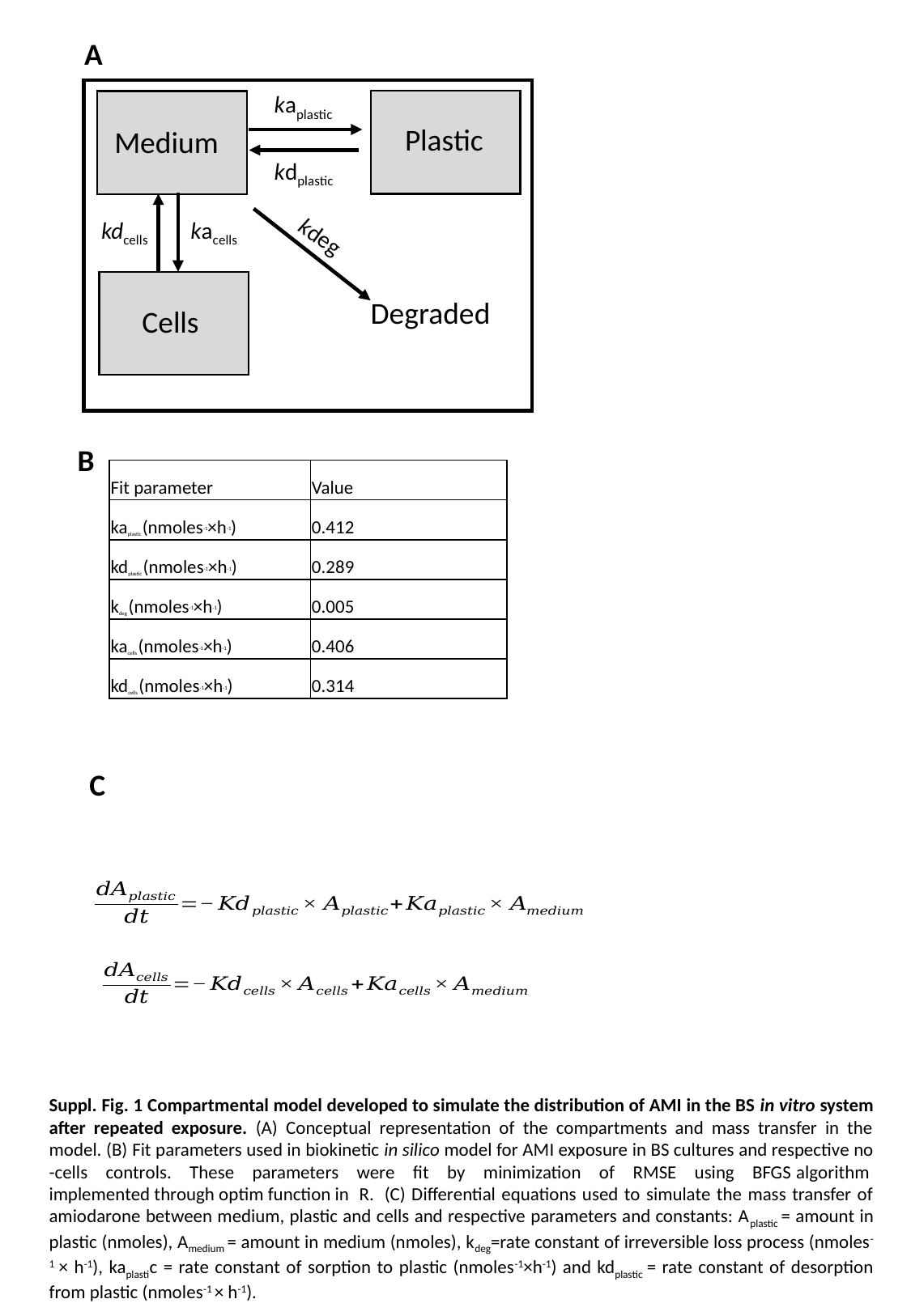

A
kaplastic
Plastic
Medium
kdplastic
kdeg
Degraded
kdcells
kacells
Cells
B
| Fit parameter ​ | Value ​ |
| --- | --- |
| kaplastic (nmoles-1×h-1) ​ | 0.412 |
| kdplastic (nmoles-1×h-1) ​ | 0.289 |
| kdeg (nmoles-1×h-1) ​ | 0.005 |
| kacells (nmoles-1×h-1) ​ | 0.406 |
| kdcells (nmoles-1×h-1) ​ | 0.314 |
C
Suppl. Fig. 1 Compartmental model developed to simulate the distribution of AMI in the BS in vitro system after repeated exposure. (A) Conceptual representation of the compartments and mass transfer in the model. (B) Fit parameters used in biokinetic in silico model for AMI exposure in BS cultures and respective no -cells controls. These parameters were fit by minimization of RMSE using BFGS algorithm  implemented through optim function in  R.  (C) Differential equations used to simulate the mass transfer of amiodarone between medium, plastic and cells and respective parameters and constants: Aplastic = amount in plastic (nmoles), Amedium = amount in medium (nmoles), kdeg=rate constant of irreversible loss process (nmoles-1 × h-1), kaplastic = rate constant of sorption to plastic (nmoles-1×h-1) and kdplastic = rate constant of desorption from plastic (nmoles-1 × h-1).

## Slide 2
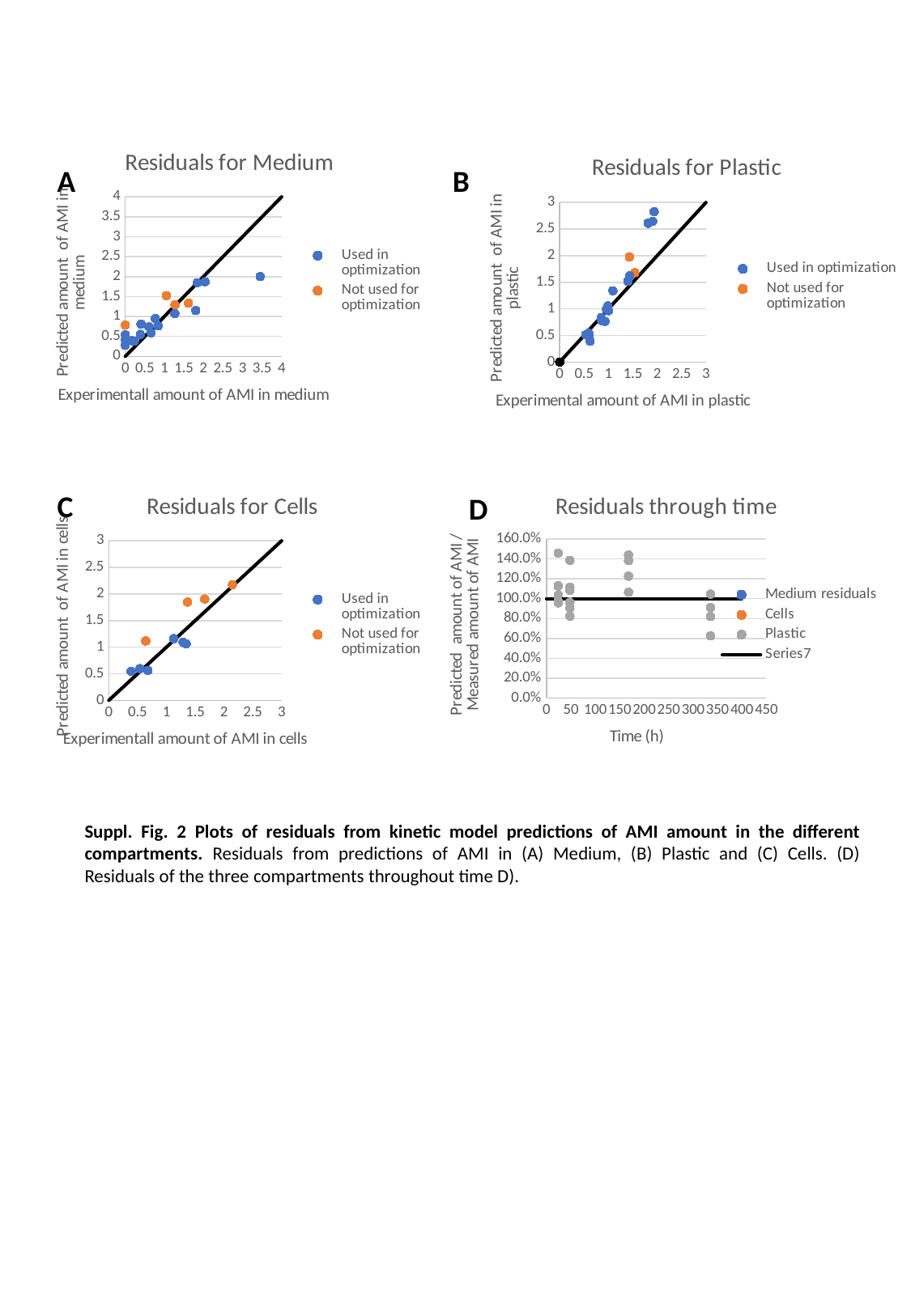

### Chart: Residuals for Medium
| Category | | | |
|---|---|---|---|
### Chart: Residuals for Plastic
| Category | | | |
|---|---|---|---|A
B
### Chart: Residuals for Cells
| Category | | | |
|---|---|---|---|
### Chart: Residuals through time
| Category | | | | |
|---|---|---|---|---|C
D
Suppl. Fig. 2 Plots of residuals from kinetic model predictions of AMI amount in the different compartments. Residuals from predictions of AMI in (A) Medium, (B) Plastic and (C) Cells. (D) Residuals of the three compartments throughout time D).

## Slide 3
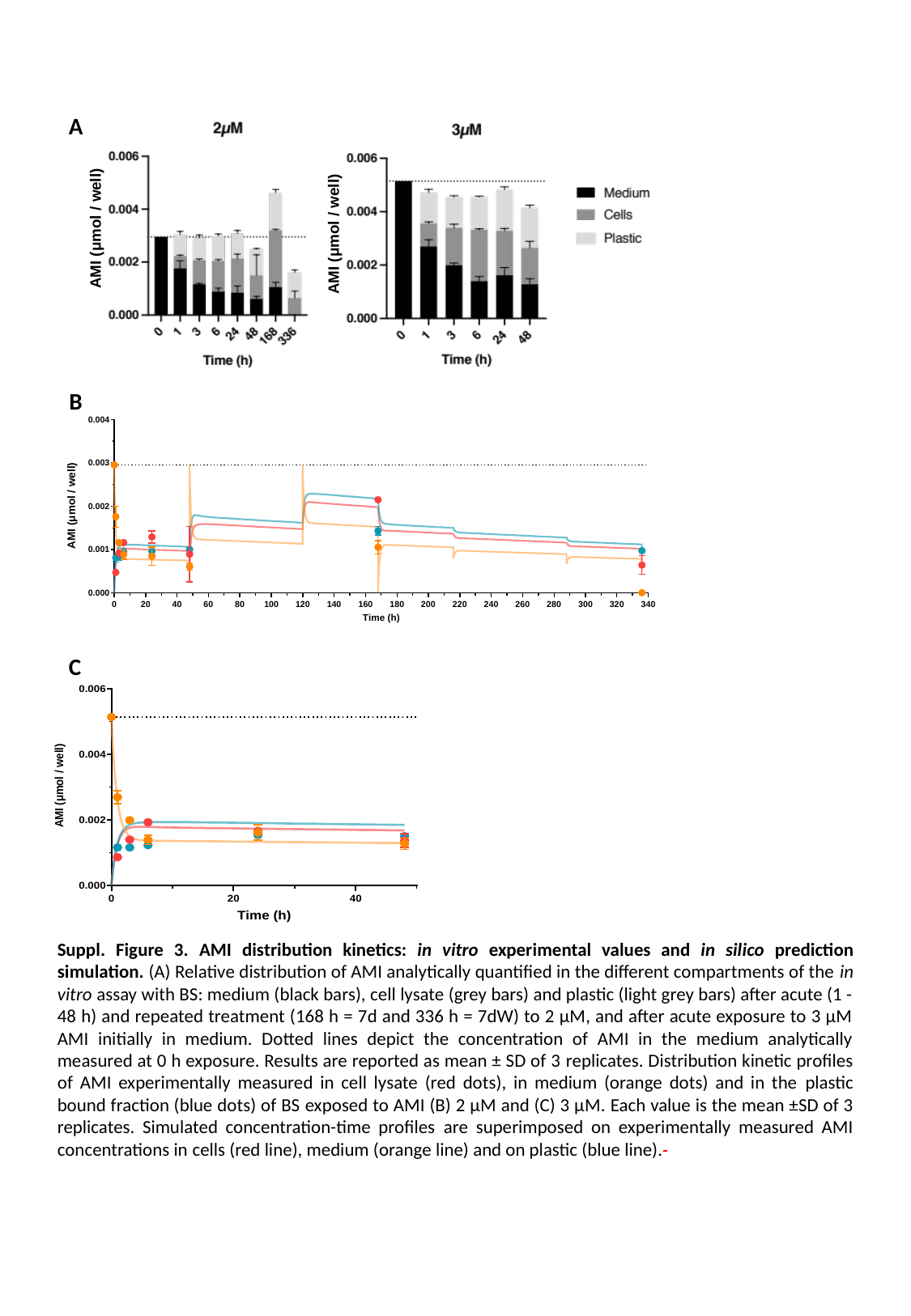

A
AMI (μmol / well)
AMI (μmol / well)
B
C
Suppl. Figure 3. AMI distribution kinetics: in vitro experimental values and in silico prediction simulation. (A) Relative distribution of AMI analytically quantified in the different compartments of the in vitro assay with BS: medium (black bars), cell lysate (grey bars) and plastic (light grey bars) after acute (1 - 48 h) and repeated treatment (168 h = 7d and 336 h = 7dW) to 2 µM, and after acute exposure to 3 µM AMI initially in medium. Dotted lines depict the concentration of AMI in the medium analytically measured at 0 h exposure. Results are reported as mean ± SD of 3 replicates. Distribution kinetic profiles of AMI experimentally measured in cell lysate (red dots), in medium (orange dots) and in the plastic bound fraction (blue dots) of BS exposed to AMI (B) 2 µM and (C) 3 µM. Each value is the mean ±SD of 3 replicates. Simulated concentration-time profiles are superimposed on experimentally measured AMI concentrations in cells (red line), medium (orange line) and on plastic (blue line).

## Slide 4
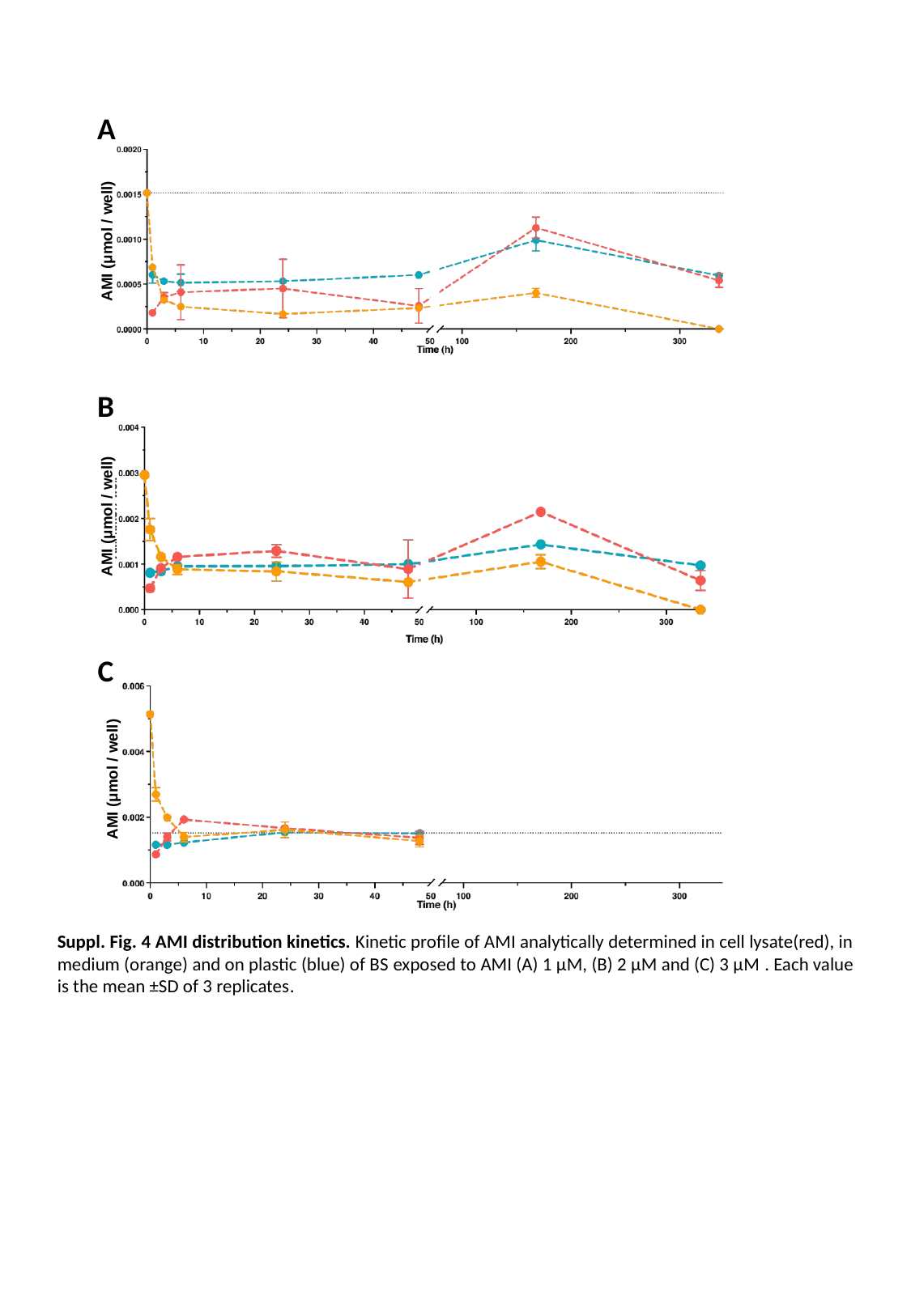

A
AMI (μmol / well)
B
AMI (μmol / well)
C
AMI (μmol / well)
Suppl. Fig. 4 AMI distribution kinetics. Kinetic profile of AMI analytically determined in cell lysate(red), in medium (orange) and on plastic (blue) of BS exposed to AMI (A) 1 µM, (B) 2 µM and (C) 3 µM . Each value is the mean ±SD of 3 replicates.

## Slide 5
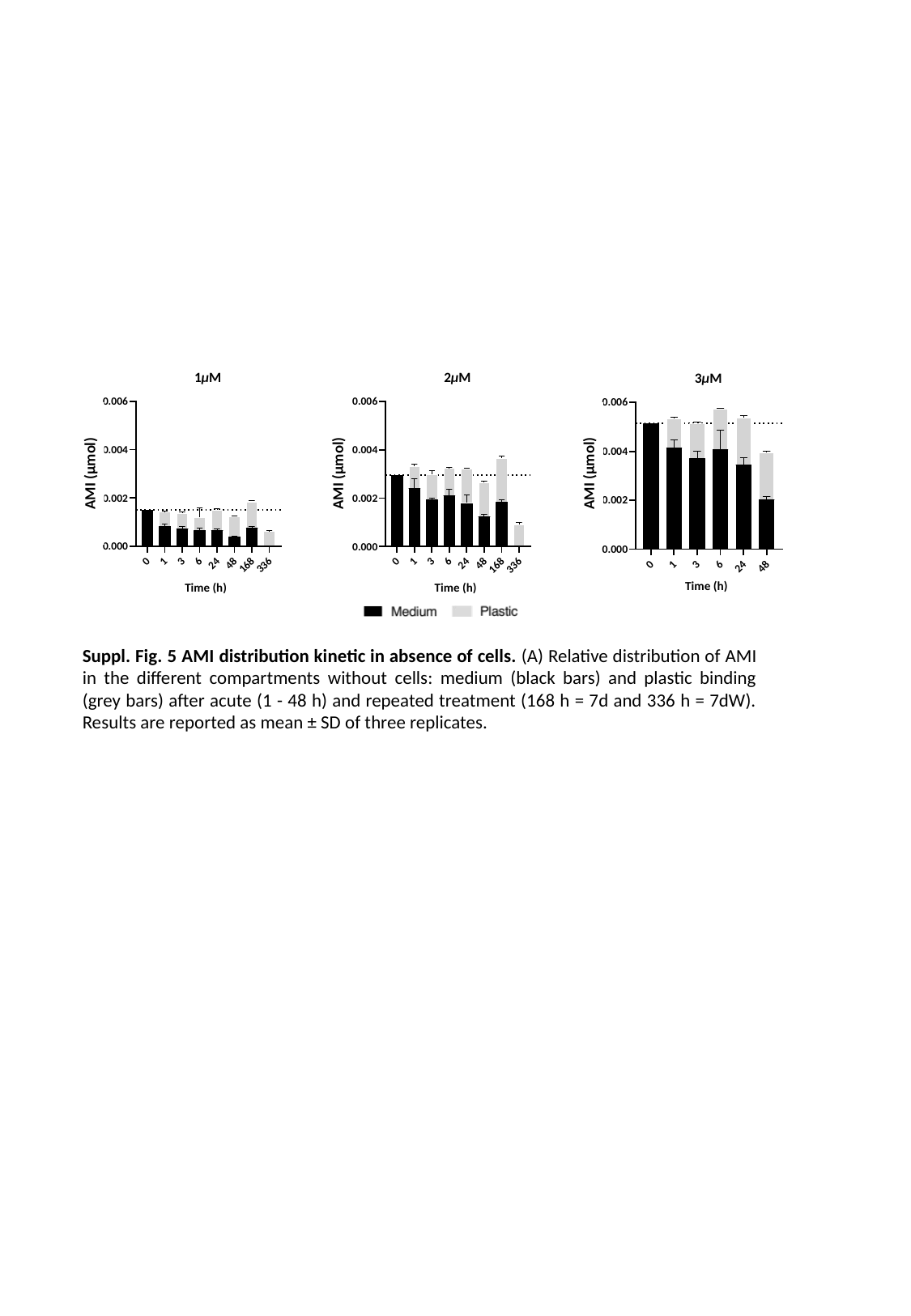

AMI (µmol)
AMI (µmol)
AMI (µmol)
Suppl. Fig. 5 AMI distribution kinetic in absence of cells. (A) Relative distribution of AMI in the different compartments without cells: medium (black bars) and plastic binding (grey bars) after acute (1 - 48 h) and repeated treatment (168 h = 7d and 336 h = 7dW). Results are reported as mean ± SD of three replicates.

## Slide 6
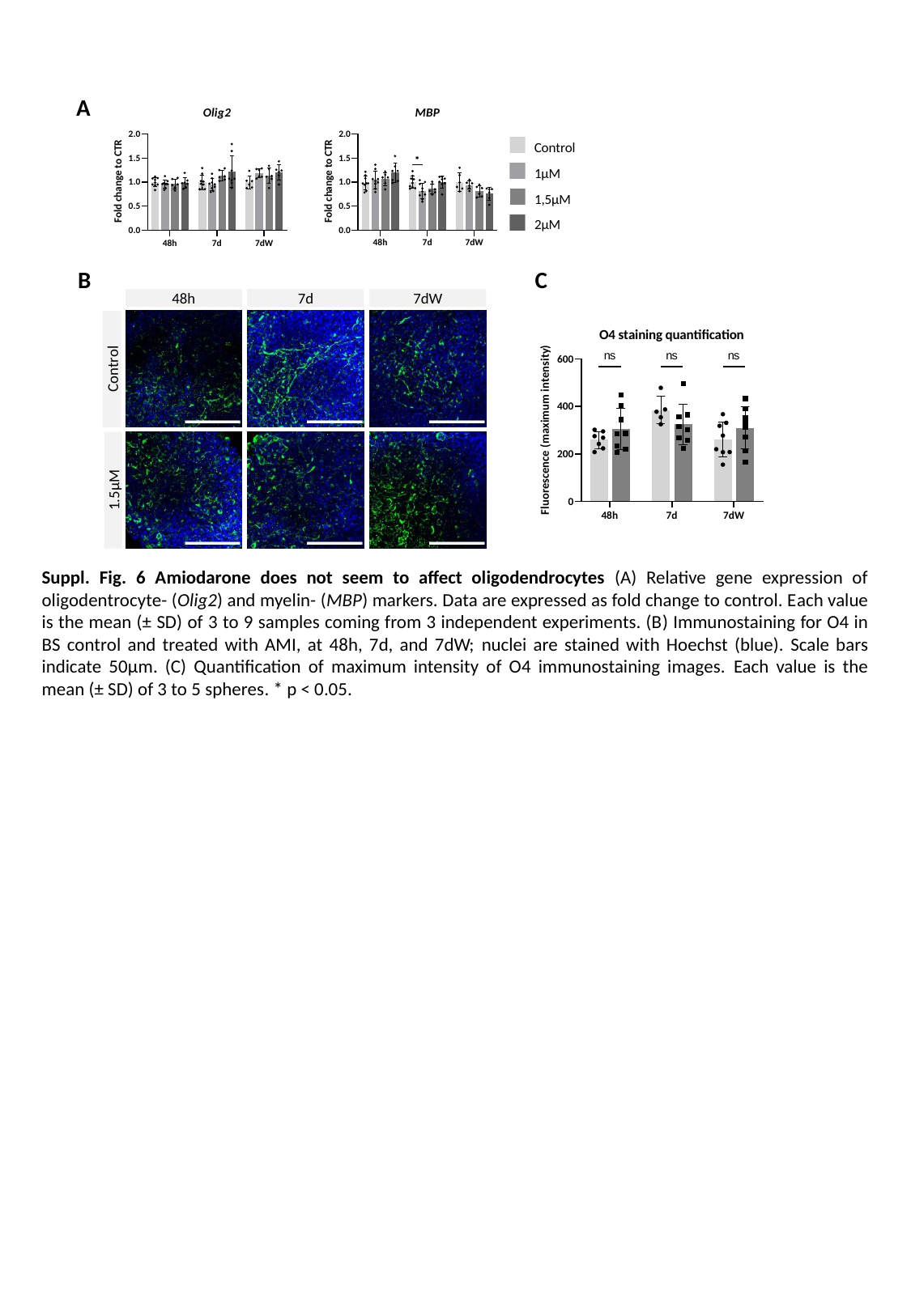

A
Control
1µM
1,5µM
2µM
B
C
48h
7d
7dW
Control
1.5µM
Suppl. Fig. 6 Amiodarone does not seem to affect oligodendrocytes (A) Relative gene expression of oligodentrocyte- (Olig2) and myelin- (MBP) markers. Data are expressed as fold change to control. Each value is the mean (± SD) of 3 to 9 samples coming from 3 independent experiments. (B) Immunostaining for O4 in BS control and treated with AMI, at 48h, 7d, and 7dW; nuclei are stained with Hoechst (blue). Scale bars indicate 50μm. (C) Quantification of maximum intensity of O4 immunostaining images. Each value is the mean (± SD) of 3 to 5 spheres. * p < 0.05.

## Slide 7
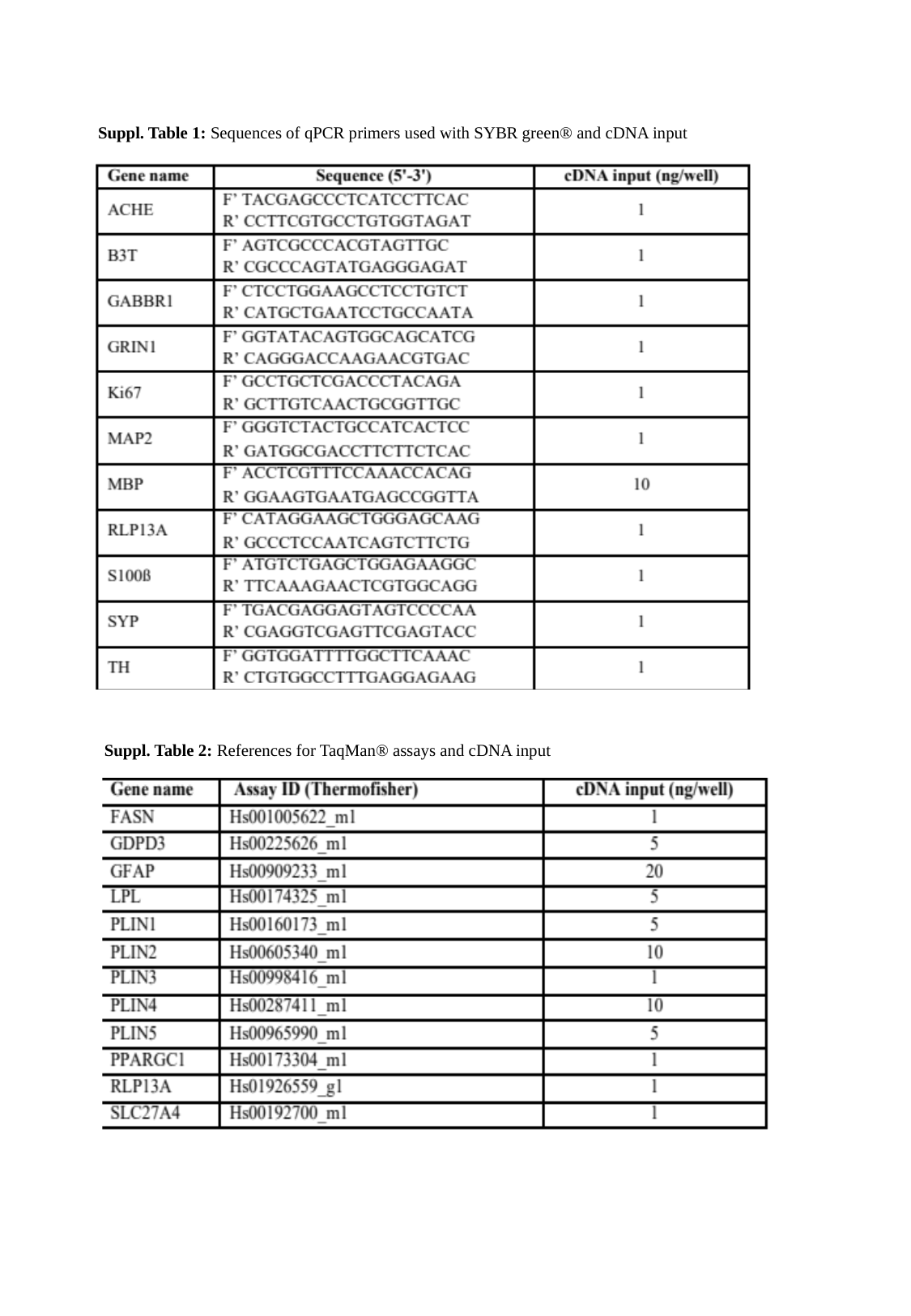

Suppl. Table 1: Sequences of qPCR primers used with SYBR green® and cDNA input
Suppl. Table 2: References for TaqMan® assays and cDNA input

## Slide 8
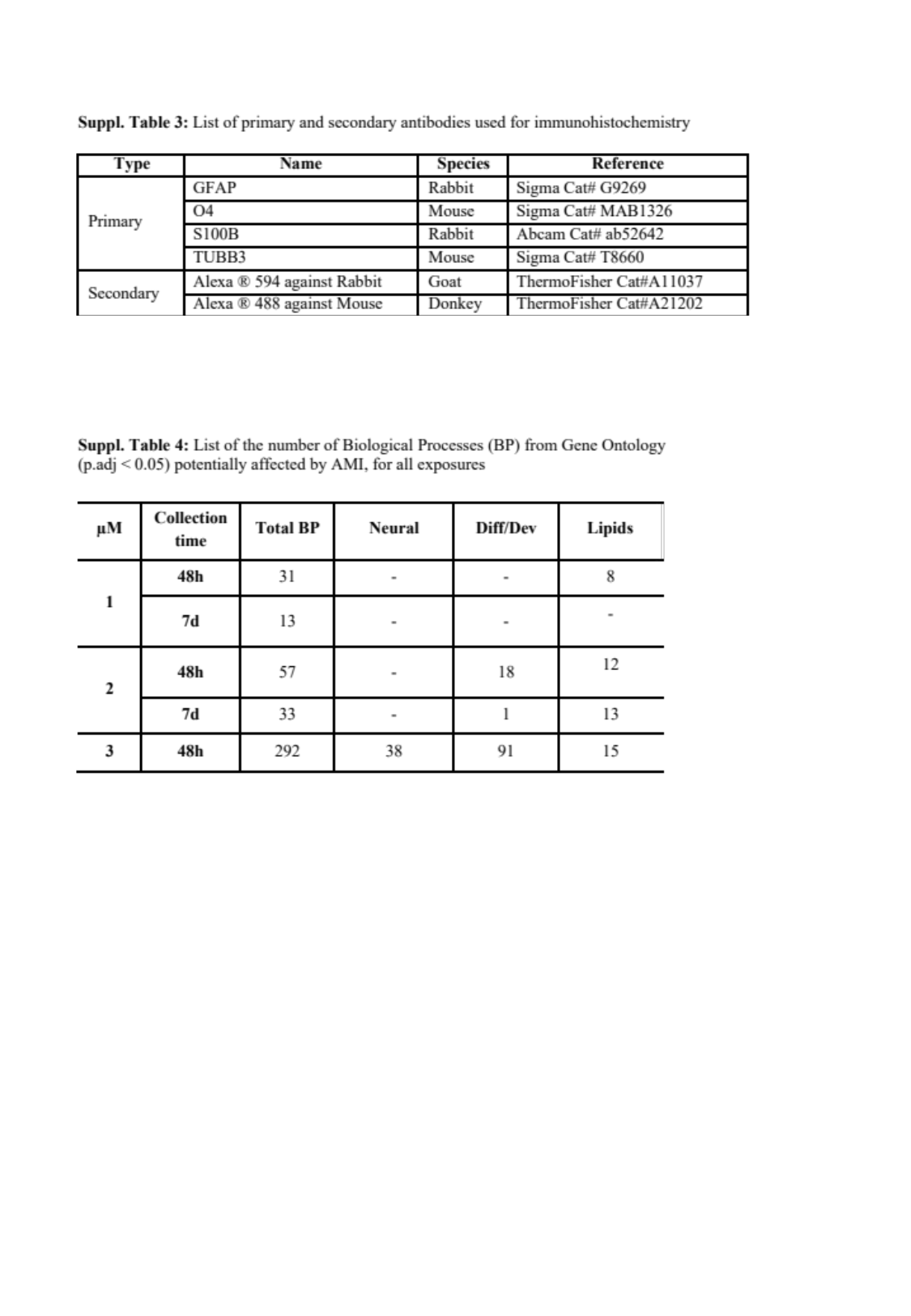

## Slide 9
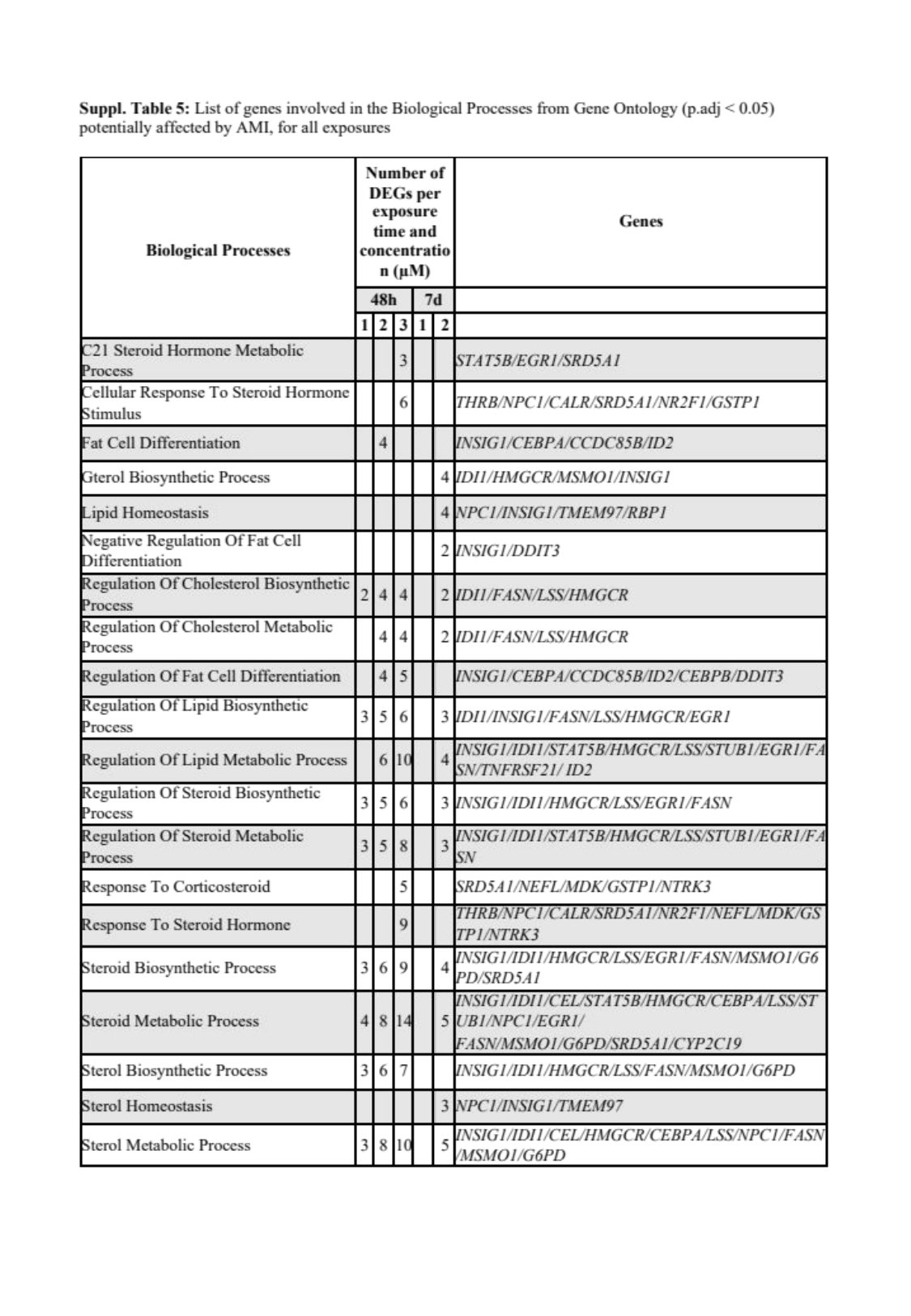

Supplement: Supplementary file 1 [file Presentation1.PPTX]
